# Lithuanian children’s trauma characteristics and correlates: comparison of clinical and non-clinical samples

**DOI:** 10.3389/fpsyt.2026.1767008

**Published:** 2026-04-24

**Authors:** Aiste Dirzyte, Ugnė Kundrotienė, Jurgita Radzevičienė, Aidas Perminas, Ina Kazakevičiūtė, Loreta Jackevičienė, Viktorija Simonavičiūtė, Milda Kumžaitė, Aleksandras Patapas

**Affiliations:** 1 Faculty of Creative Industries, Vilnius Gediminas Technical University, Vilnius, Lithuania; 2 Institute of Psychology, Mykolas Romeris University, Vilnius, Lithuania; 3 Department of Psychology, Klaipeda University, Klaipeda, Lithuania; 4 Department of Children Psychiatry, Vilnius University Hospital Santaros Klinikos, Vilnius, Lithuania; 5 Department of Psychology, Vytautas Magnus University, Kaunas, Lithuania; 6 Institute of Public Administration, Mykolas Romeris University, Vilnius, Lithuania

**Keywords:** dissociation, exposure to potentially traumatic events, mood, post-traumatic cognitions, post-traumatic stress

## Abstract

**Introduction:**

Previous studies have shown that children’s exposure to potentially traumatic events and their trauma−related symptoms may not always be consistently identified. This study aims to examine differences in trauma exposure and related psychological outcomes between clinical and non−clinical Lithuanian children.

**Methods:**

This cross-sectional study included 10–17−year−old children and adolescents recruited from a clinical inpatient setting (Vilnius University Hospital Santaros Klinikos) and general−education schools in Vilnius and nearby districts. After parental consent and child assent, participants completed a secure mobile assessment covering exposure to potentially traumatic events (CATS), dissociation (A−DES), mood and feeling (SMFQ), post−traumatic cognitions (CPTCI), PTSD symptoms (CATS; PCL−5 for convergent validation), and perceived social support (CASSS). Data were collected in 2023–2024. Group differences were examined using Welch’s t−tests (with Mann–Whitney U as robustness checks), and associations were assessed using Pearson correlations.

**Results:**

In the clinical sample over 40% of children experienced physical violence, while in the non−clinical sample 82.9% children reported exposure to multiple traumatic events. The clinical sample showed significantly higher dissociation, negative mood, and PTSD symptoms compared to the non−clinical sample. However, among children exposed to more than one traumatic event, differences in dissociation, PTSD symptoms, and close−friend support were not significant. Across both samples, exposure to potentially traumatic events was strongly associated with PTSD symptoms, dissociation, and post−traumatic cognitions, and moderately associated with mood symptoms. In the non−clinical sample, parental support showed moderate negative associations with dissociation, mood symptoms, post−traumatic cognitions, and PTSD symptoms.

**Discussion:**

This study identified between−sample differences in exposure to potentially traumatic events and trauma−related psychological outcomes among Lithuanian children in inpatient and community settings, highlighting the need for trauma−informed assessment and attention to social support within child mental health and welfare services.

## 1 Introduction

Exposure to potentially traumatic events (PTEs) such as war, abuse, natural disasters, or the loss of loved ones is associated with psychological outcomes (1–3) such post−traumatic stress, anxiety, aggression, dissociation, cognitive and social impairments, low self−esteem, and risk behaviors (4–7). A meta−analysis indicated that trauma has been associated with higher odds of depression by 2.6 times (8). In 2022, about 469 million children lived in conflict−affected areas (9), with many more exposed to hunger or abuse, which is associated with elevated mental−health risks (10). Similar patterns are described in recent nursing research showing that child abuse and neglect can lead to persistent emotional distress, anxiety, and trauma−related symptoms (11). Evidence from conflict−affected regions, such as Kashmir, shows that children living in unstable environments frequently report persistent PTSD symptoms and benefit from targeted identification and care (12). Even outside crisis contexts, children may face multiple stressors (Sachser, C 2022), underscoring the need to understand resilience factors (2, 13, 14) and the cumulative long−term effects of trauma (14–16). Trauma−exposed youth also commonly experience social withdrawal and peer−relationship difficulties, which may be associated with reduced socioemotional development (17–21). Repeated exposure and the absence of emotional support are particularly associated with adverse outcomes (22).

Population−level data point to exceptionally high rates of potentially traumatic events among Lithuanian adolescents. In a survey of 1,299 adolescents, 71.9% reported at least one traumatic event; half reported emotional abuse, one third physical abuse, and one fifth neglect (23–25). Despite this prevalence, assessment of children’s traumatic experiences and trauma−related symptoms in Lithuanian clinical settings remains inconsistent and fragmented. Unrecognized and unaddressed experiences have been associated with long−term difficulties, such as mistrust, low self−esteem, self−harm tendencies, heightened reactivity to stress, somatic complaints, sleep disturbance, enuresis/encopresis, aggressive or risky behavior, addiction, and later criminal activity (26, 27). Early identification is therefore critical, as timely detection of psycho−traumatization may be associated with reduced adverse developmental and mental−health outcomes (14). These findings illustrate how exposure to violence, instability, and inadequate protection can intensify children’s vulnerability to trauma−related difficulties. Although research has expanded knowledge about the psychological outcomes of PTE exposure (2, 13, 28, 29), differences between clinically hospitalized and general−population children remain under−explored, and unrecognized trauma may be associated with a higher risk of misdiagnosis and inadequate treatment, with consequences persisting into adulthood (30).

Dissociation and trauma−related cognitions—are central to understanding which children exhibit more severe post−traumatic reactions. Dissociation, a disruption in normally integrated consciousness, memory, identity, and perception, may be understood as a response pattern associated with attempts to regulate intolerable affect but is associated with difficulties in memory integration and adaptive processing (31, 32). In children, dissociation can manifest as depersonalization, derealization, amnesia, identity confusion, flashbacks, and concentration problems; its expression varies by trauma characteristics, developmental stage, and contextual support, with potential contributions from genetic/neurological vulnerability and early attachment disruptions (5, 33–40). Post−traumatic cognitions constitute a second mechanism. After trauma, children may endorse self−blame, see themselves as damaged, and view the world as dangerous and others as untrustworthy (1, 4, 7, 41–43). Such beliefs are closely related to symptom severity and are core treatment targets (6, 44). Together, dissociation and maladaptive cognitions align with persistent negative moods—sadness, irritability, and anxiety—often sustained by intrusions and avoidance (45–52).

Post−traumatic stress symptoms (PTSS) denote distress and functional impairment following exposure to overwhelming events in youth (53). Symptom clusters include re−experiencing (intrusions, nightmares, flashbacks), avoidance of reminders, hyperarousal and reactivity, and negative alterations in cognitions and mood, including persistent negative affect and reduced positive emotions (54–57). Not all trauma−exposed children develop PTSS, and responses vary with risk and protective factors (58). Heightened emotions such as fear, sadness, and anger may remain elevated following the event and are associated with poorer concentration and school performance (48, 59). Dissociation may also occur, which is associated with detachment and memory disruptions (5, 40, 60). These patterns highlight the value of monitoring mood and cognition when assessing trauma−related distress in children (46, 61).

Perceived social support is a key contextual factor, yet it is often treated as a single construct, obscuring distinct functions of parents, teachers, and peers (62). Reviews suggest that family support shows a relatively stable association with lower PTSS, whereas teacher support can be especially salient when caregivers are distressed or unavailable (62–65). In school contexts, teachers may act as quasi−attachment figures, offering safety cues and co−regulation in daily routines (66). Findings for peer support are mixed, indicating the need to differentiate sources when evaluating links with dissociation, cognitions, mood, and PTSS within the same study (17, 62, 63, 65, 67, 68).

Although research has examined trauma’s effects on emotional, cognitive, behavioral, and social functioning (1, 5, 13, 28, 29, 69–71), comparative knowledge about trauma−related processes in children hospitalized in psychiatric clinics versus those in the general population remains limited, particularly in Lithuania. This gap matters because clinical samples often include children with complex presentations whose trauma histories have not been systematically assessed. Unaddressed experiences may be associated with functioning into adulthood (30). Youth with post−traumatic stress symptoms frequently show impaired social functioning (72), and the co−occurrence of PTSD with chronic pain or sleep disturbance is associated with greater impairment and elevated suicide−risk indicators (73). These observations warrant an integrated examination of trauma−related symptoms, cognitive−affective mechanisms, and social resources across both clinical and non−clinical samples within the Lithuanian context.

This study aims to examine differences exposure to potentially traumatic events (PTEs) and psychological outcomes between clinical and non-clinical samples of Lithuanian children. In accordance with trauma research standards, we distinguish between exposure to potentially traumatic events (PTEs) and their psychological outcomes (PTSD symptoms, mood changes, dissociation post−traumatic cognitions, and perceived social support). By examining these constructs concurrently, the study seeks to clarify how trauma−related psychological outcomes cluster within these two samples. Based on prior literature, the study tests four *a priori* hypotheses (see **Table 1**).

**Table 1:** Summary of study hypotheses.

Hypothesis	Core idea	Variables	Expected direction
H1	Group differences between clinical and non−clinical sample	Exposure to PTEs; impairment in psychosocial functioning; CATS PTSD symptoms; dissociation; mood symptoms; PTSD Checklist symptoms; post−traumatic cognitions; perceived social support.	Clinical > non−clinical for symptoms, Non−clinical > clinical for perceived social support
H2	Group differences between clinical sample and non−clinical sample exposed to >1 PTE	Same variables as H1, but focusing on the non−clinical ≥1 PTE subsample	Clinical > non−clinical >1 PTE for symptom severity; Non−clinical > clinical for perceived social support
H3	Trauma-related mechanisms	Exposure to PTEs will be associated with higher levels of PTSD symptoms, dissociation, negative mood, and post-traumatic cognitions.	Higher PTE exposure → higher symptoms (association only)
H4	Role of social support	Perceived social support from parents, teachers, and peers will be associated with lower trauma-related psychological outcomes, though patterns may differ across groups.	Higher support → lower psychological outcomes (PTSD symptoms, dissociation, mood, post−traumatic cognitions) (association only)

## 2 Materials and methods

### 2.1 The sample

The study employed two distinct samples—a clinical and a non−clinical sample—to align with the objectives of the national biomedical project aimed at improving the assessment of traumatic experiences among Lithuanian children. For this study, the clinical sample was operationally defined as children and adolescents (10–17 years) hospitalized in a child psychiatry department and receiving clinical assessment and treatment at the time of recruitment; the non−clinical group was defined as children and adolescents (10–17 years) recruited from general−education schools who were not receiving psychiatric care. The clinical sample represents children hospitalized in a child psychiatry department, where trauma exposure, emotional dysregulation, and complex presentations are highly prevalent, and where validated trauma−assessment tools are needed for accurate identification and treatment planning. The non−clinical sample provides a normative comparison sample that is essential for distinguishing clinically significant patterns from population−level variation, as Lithuania currently lacks standardized reference data for trauma exposure and trauma−related psychological outcomes. The inclusion of both samples allows for examining differences in symptom severity, cognitive−affective mechanisms, and perceived social support.

The study used a cross-sectional design. The whole study sample (n=332) consisted of clinical (n=50) and non-clinical (n=282) participants. Because this dataset is part of an ongoing national biomedical research project, the sample size was determined by the number of eligible participants available during the recruitment period rather than by an *a priori* power analysis.

The sociodemographic and other characteristics of participants at baseline in clinical and non-clinical samples are presented in **Table 2**.

**Table 2:** Characteristics of participants at baseline in clinical and non-clinical samples.

Baseline characteristic	Clinical sample (*n* = 50)		Non-clinical sample (*n* = 282)	
	*n*	%	*n*	%
Gender				
Female	26	52.0	184	65.2
Male	20	40.0	98	34.8
Prefer not to answer	4	8.0	0	0
	**M**	**SD**	**M**	**SD**
Age	12.98	1.918	13.64	2.096
Female	13.54	1.860	13.87	2.131
Male	12.25	1.888	13.22	1.972
Prefer not to answer	n = 2			

M, Mean; SD, standard deviation.

The clinical participants were recruited from the Child Psychiatry Department of Vilnius University Hospital Santaros Klinikos. Eligible participants were hospitalized children aged 10–17 years who were capable of completing self−report measures. Children with severe cognitive or developmental impairments preventing assessment were not included. The clinical sample was heterogeneous, reflecting common inpatient child−psychiatry presentations; their ICD−10 diagnoses are presented in **Table 3**. Notably, none of the clinical participants had a formal diagnosis of PTSD (F43.1). As PTSD was assessed through self−report only, scores were interpreted as indicating screening−level PTSD rather than confirmed diagnoses. No structured diagnostic interview (PLC-5) was conducted. No screening of trauma types was conducted prior to assessment, as the purpose of the study was to capture the natural variability in children’s trauma histories rather than restrict analyses to predefined trauma categories.

**Table 3:** Diagnoses of participants of the clinical sample.

	Clinical sample (*n* = 50)	
Diagnosis *	*n*	%
F23.10	1	2.44
F23.80	2	4.88
F32.10	9	21.95
F32.30	1	2.44
F50.1	1	2.44
F50.8	1	2.44
F51.2	1	2.44
F81.3	6	14.63
F84.0	3	7.32
F84.5	2	4.88
F90.0	1	2.44
F91.8	3	7.32
F92.0	1	2.44
F93.8	20	48.78
F95.0	1	2.44
R45.81	11	26.83
R63.3	1	2.44
Z61.0	1	2.44
Z61.4	1	2.44
Z61.8	9	21.95
Z62.8	2	4.88
Z63.8	3	7.32
Z71.3	1	2.44
Z73.1	4	9.75
Z91.5	8	19.51

*Categories are not exclusive.

The non−clinical sample was recruited from several Lithuanian schools in Vilnius and nearby districts. Approximately 1500 students and their parents were invited, of whom 282 agreed to participate (~19% response rate). The clinical group demonstrated a considerably higher response rate (50 out of ~60 invited).

Across both groups, inclusion criteria required: (a) age 10–17 years, (b) parental/legal−guardian informed consent, and (c) child assent; exclusion criteria included inappropriate age (<10 or >17 years). Participants and their caregivers were informed about the study aims, voluntary participation, confidentiality, and the right to discontinue participation at any time. No parent-related variables (such as parental education, occupation, or income) were collected.

### 2.2 Consideration of age, cognitive, and linguistic capacities

The study included children aged 10–17 years because the validated trauma−assessment instruments used in this project were developed for children within this developmental range. Children younger than 10 years or those with cognitive or developmental difficulties that would prevent them from reliably completing self−report measures were not included. All questionnaires were administered using age−appropriate language, and younger participants were supported by trained psychologists to ensure comprehension. These procedures helped minimize the influence of developmental differences in cognitive or linguistic capacities on trauma reporting.

### 2.3 Procedure

Data collection for the clinical sample began in July 2023. Eligible inpatients and their parents were approached on the day of hospitalization and asked to participate. In the clinical sample, recruitment was conducted by trained clinical staff. Data collection for the non−clinical sample began in September 2023 in schools. After obtaining parental consent and child assent, participants completed questionnaires via a secure mobile application specifically developed for the broader biomedical project. In the non-clinical sample, recruitment was conducted by psychologists in quiet and private rooms. Written informed consent from parents/guardians and written assent from children were obtained prior to any procedures. Participants received written and verbal information, had opportunities to ask questions, and then signed informed consent forms. Both forms outlined aims, procedures, risks, benefits, confidentiality, data protection, the right to decline or withdraw without consequences, and contacts for ethics and data−protection queries. A psychologist was available during/after survey completion to address any distress; participants could withdraw at any time. Completion took approximately 60 minutes. Data for both clinical and non−clinical samples were collected online between July 3, 2023 and December 24, 2024.

The study was conducted as part of a larger biomedical research project “Assessment of Children’s Traumatic Experiences and Implementation of TF−CBT in Lithuania”, approved by the Vilnius Regional Biomedical Research Ethics Committee (approval No. 2023/4−1499−963).

### 2.4 Measurements

*Child and Adolescent Trauma Screen (CATS)* was applied to assess children’s traumatic experiences (6, 74, 75. *CATS* measures 1) potentially traumatic events, 2) posttraumatic stress symptoms, and 3) impairment in psychosocial functioning according to DSM-5 and ICD-11 criteria in children and adolescents aged from 7 to 17 years. Consistent with our terminology, CATS PTE checklist indexes exposure (events), whereas CATS PTSS and impairment index psychological outcomes. The potentially traumatic events checklist includes 15 items assessing experiences of natural disasters, serious accidents, violence at home or in the community, sexual abuse (off- and online), bullying, cyberbullying, traumatic loss, medical procedures, and war. Respondents can reveal whether they had experienced the event by checking ‘yes’ or ‘no’. Second, posttraumatic stress symptoms in the last four weeks are then assessed using 20 items rated on a 4-point Likert scale with the following anchors: 0 = ’Never’, 1 = ’Sometimes’, 2 = ’Often’, 3 = ’Almost Always’. Third, impairment in psychosocial functioning is assessed via five ‘yes’ or ‘no’ items that ask whether the previously rated posttraumatic stress symptoms interfere with five key areas of functioning (getting along with others, school/, hobbies, relationships, and general happiness). Previous studies reported a reliability range between 0.88 and 0.94, and the convergent-discriminant validity pattern showed medium to strong correlations with measures of depression and anxiety (6, 74, 76–78).

*The Adolescent Dissociative Experience Scale- II (A-DES)* was used to assess dissociative symptoms in clinical and non-clinical samples (34, 35). A−DES → “assesses a psychological outcome (dissociation)”. The 30 items in the A-DES are rated on a scale of 0 (“never”) to 10 (“always”) based on children’s self-report symptoms. The total A-DES score is the mean of the 30-item scores. A-DES has been validated in previous studies and demonstrated high reliability (33–35).

*Short Moods and Feelings Questionnaire (SMFQ)* was utilized to compare children’s moods and feelings in clinical and non-clinical samples (61). SMFQ → “assesses a psychological outcome (mood symptoms)”. The SMFQ is a short measure of symptoms of depression in children and adolescents and is validated for use in children aged 6 years and up. The self-report version of the SMFQ, used for this study, consists of 13 items measured on a 3-point Likert scale with 0 = ’Not true’, 1 = ’Sometimes’, and 2 = ’True’ (range 0–26). The SMFQ has been validated with several samples and demonstrated a high reliability of up to 0.91 (46, 61, 79, 80).

*PTSD Checklist for DSM-5 (PCL-5)* was employed to assess children’s post-traumatic stress symptoms (81–83). PCL−5 → “assesses a psychological outcome (PTSD symptoms)”. Post−traumatic stress symptoms were assessed with the PTSD Checklist for DSM−5 (PCL−5). PCL-5 is a 20-item self-report measure that assesses 20 symptoms of PTSD as outlined in the DSM-5, organized into four symptom clusters: ‘Intrusion symptoms’ (items 1–5), ‘Avoidance’ (items 6–7), ‘Negative alterations in cognition and mood’ (items 8–14), and ‘Alterations in arousal and reactivity’ (items 15–20). Respondents indicate how much they were bothered by a symptom on a 5-point Likert scale ranging from ‘Not at all’ (0) to ‘Extremely’ (4). The probability of PTSD is verified by endorsing symptoms at ‘Moderately’ (2) or above for at least one ‘Intrusion’ and ‘Avoidance’ symptom, and two ‘Negative alterations in cognition and mood’ and ‘Alterations in arousal and reactivity’ symptoms. The validity of the PCL-5 was supported by prior studies which revealed the high reliability of 0.93 of the instruments (82, 84, 85).

*Children’s Post-Traumatic Cognitions Inventory (CPTCI)* was used to assess children’s cognitions in clinical and non-clinical samples (4). In this study, CPTCI indexes a psychological outcome (post-traumatic cognitions) rather than exposure. The CPTCI is a self-report scale with 25 items, which aims at assessing negative posttraumatic appraisals in children and adolescents aged 6 to 17 years old on a Likert scale ranging from 1 (don’t agree at all) to 4 (agree a lot). The CPTCI includes components of a sense of “permanent and disturbing change” and a sense of being a “fragile person in a scary world.” Previous studies reported good internal consistency (Cronbach’s α values between.86 and.96) and moderate correlations with measures of depression and post-traumatic symptoms (4, 41, 86–88).

*The Child and Adolescent Social Support Scale (CASSS)* was used to assess children’s and adolescents’ perceived social support (89). CASSS is a 40−item, multidimensional measure indexing perceived support from four sources: parents, teachers, classmates, and close friends (10 items per subscale). In this study, the Level 1 version of the CASSS was administered to all participants. Study participants were asked to respond to statements such as “My parent(s) give me good advice,” “My teacher(s) understand me,” “My classmates ask me to join activities,” and “My close friend understands my feelings”. Responses were recorded on a 6−point frequency scale from 1 = Never to 6 = Always. Subscale scores are computed by summing the 10 items corresponding to each source, and a Total social support score is obtained by summing all four subscales. Total scores therefore range from 40 to 240, with higher scores indicating greater perceived social support. Psychometric evidence reported in the original publication indicates strong internal consistency and support for the intended factor structure. Specifically, Total scale reliability is approximately α = .94 (Level 1) and α = .95 (Level 2), and subscale alphas typically fall in the.87–.94 range. In addition, confirmatory factor analyses (CFA) support a four−source structure (Parent, Teacher, Classmate, Close Friend) with acceptable model−fit indices (e.g., CFI/NNFI ≳.90, RMSEA ≈.05). Additional studies using the CASSS further support its utility across school−age samples and emphasize the practical value of frequency and importance ratings (90, 91).

**Table 4** presents Cronbach’s α and McDonald’s ω values that show the internal consistency regarding the scales used in this research in clinical and non-clinical samples.

**Table 4:** Cronbach’s α and McDonald’s ω values of the scales used in clinical and non-clinical samples.

Scales	Variables	Cronbach’s α		McDonald’s ω	
		Clinical	Non-clinical	Clinical	Non-clinical
*CATS*	Posttraumatic stress symptoms	0.934	0.950	0.937	0.951
*A-DES*	Dissociation symptoms, overall	0.956	0.960	0.958	0.961
*SMFQ*	Moods and feelings, overall	0.901	0.931	0.902	0.933
*PCL-5*	PTSD Checklist, overall	0.940	0.962	0.942	0.963
	Intrusion symptoms	0.858	0.901	0.859	0.903
	Avoidance	0.757	0.835	0.757	0.835
	Negative alterations in cognition and mood	0.847	0.897	0.860	0.901
	Alterations in arousal and reactivity	0.860	0.872	0.864	0.874
*CPTCI*	Post-Traumatic Cognitions, overall	0.947	0.951	0.948	0.952
	Permanent and disturbing change	0.890	0.927	0.893	0.928
	Fragile person in a scary world	0.888	0.889	0.890	0.891
*CASSS*	Social support, overall	0.918	0.965	0.918	0.965
	Parental support	0.949	0.969	0.951	0.970
	Teacher support	0.923	0.944	0.925	0.945
	Classmates support	0.929	0.962	0.930	0.963
	Close Friend support	0.918	0.956	0.921	0.957

For the statistical analyses, the SPSS (version 29) software and JASP (version 0.16.04.0) software were applied. To test the reliability of the instruments, Cronbach’s α and McDonald’s ω were assessed. Next, the normality of data distribution was evaluated (Shapiro-Wilk test, skewness, and kurtosis). Independent samples’ T-test was applied to identify the distinctions in traumatic experiences, dissociative symptoms, mood symptoms, post-traumatic stress symptoms, and post-traumatic cognitions in clinical and non-clinical samples. Correlation analyses were conducted to examine the associations between the study variables separately in clinical and non-clinical samples.

## 3 Results

### 3.1 Data distribution in the clinical and non-clinical samples

In the clinical and non-clinical samples, the Shapiro-Wilk test was mainly significant, but the skewness and kurtosis suggested that the data may be considered normally distributed as most of the data fell within the range of +/-2 (92), as demonstrated in **Table 5**, so the parametric statistics could be applied.

**Table 5:** Data distribution in the clinical and non-clinical samples.

Scales	Variables	Clinical Sample			Non-clinical Sample		
		W	S	K	W	S	K
*CATS*	Potentially traumatic events	0.926*	0.948	0.734	0.895***	1.276	2.272
	Posttraumatic stress symptoms	0.982	0.102	-0.700	0.936***	0.708	-0.259
	Impairment in psychosocial functioning	0.846***	-0.587	-0.905	0.856***	0.482	-1.134
*A-DES*	Dissociation symptoms, overall	0.942*	0.734	-0.156	0.908***	0.978	0.267
*SMFQ*	Moods and feelings, overall	0.951	-0.259	-0.956	0.912***	0.749	-0.456
*PCL-5*	PTSD Checklist, overall	0.966	-0.272	-0.770	0.873***	0.980	-0.017
	Intrusion symptoms	0.948*	0.146	-0.838	0.824***	0.979	-0.354
	Avoidance	0.911**	0.107	-1.356	0.822***	0.741	-0.797
	Negative alterations in cognition and mood	0.946*	-0.382	-0.916	0.880***	0.932	0.091
	Alterations in arousal and reactivity	0.955	-0.116	-0.956	0.871***	1.049	0.361
*CPTCI*	Post-Traumatic Cognitions, overall	0.980	-0.169	-0.756	0.923***	0.859	0.052
	Permanent and disturbing change	0.956	0.094	-1.034	0.832***	1.257	0.829
	Fragile person in a scary world	0.962	-0.510	-0.141	0.967***	0.377	-0.646
*CASSS*	Social support, overall	0.987	-0.248	-0.149	0.957***	-0.830	0.940
	Parent support	0.944	-0.668	-0.105	0.892***	-0.969	0.233
	Teacher support	0.988	0.043	-0.240	0.968***	-0.371	-0.610
	Classmates support	0.965	0.570	0.174	0.960***	-0.449	-0.580
	Close Friend support	0.899**	-1.225	1.534	0.835***	-1.466	1.909

*W, Shapiro-Wilk test*; *S*, skewness; *K*, kurtosis; *p <.05, **p <.01, ***p <.001.

The frequency of experiences of exposure to potentially traumatic events (PTEs) in clinical and non-clinical samples is presented in **Table 6**. More than 40 percent of participants in the clinical sample revealed that they were slapped, punched, or beaten up in their family, or saw someone being slapped or punched, or someone close to them died suddenly, or they experienced other scary events. In the non-clinical sample, 82.9 percent of children disclosed more than one PTE.

**Table 6:** Frequency of experiences of PTEs in clinical and non-clinical samples. .

Potentially traumatic events	Clinical sample (*n* = 50)		Non-clinical sample (*n* = 282)	
	*n (yes)*	%	*n (yes)*	%
1. Serious natural disasters like a flood, hurricanes, earthquakes, or fires.	15	30.6	34	12.1
2. Serious accident or injury like a car/bike crash, dog bite, sports injury.	17	34.0	99	35.2
3. Robbed by threat, force, or weapon.	6	12.0	17	6.0
4. Slapped, punched, or beat up in your family.	21	42.0	47	16.7
5. Slapped, punched, or beaten up by someone not in your family.	18	36.0	74	26.2
6.Seeing someone in your family get slapped, punched, or beat up.	18	36.0	38	13.5
7. Seeing someone in the community get slapped or punched.	22	44.0	91	32.3
8. Someone older touching your private parts when they shouldn’t.	13	26.5	44	15.6
9. Someone forcing or pressuring sex, or when you couldn’t say no.	4	8.2	18	6.4
10. Someone close to you dies suddenly or violently.	23	46.9	111	39.4
11. Attacked, stabbed, shot at, or hurt badly.	6	12.2	14	5.0
12. Seeing someone attacked, stabbed, shot at, hurt badly, or killed	8	16.3	37	13.1
13. Stressful or scary medical procedure.	18	36.7	75	26.7
14. Being around war.	3	6.1	6	2.1
15. Other stressful or scary events.	25	52.1	78	27.8

### 3.2 Analysis of differences between clinical and non-clinical samples

To test hypothesis H1, an independent samples t−test was applied. Because Shapiro–Wilk indicated departures from normality, we used Welch’s t−tests as primary analyses (the sample sizes were unequal) and conducted Mann–Whitney U tests as robustness checks. The means, standard deviations, and results for the clinical and non−clinical samples are presented in **Table 7**.

**Table 7:** Means, standard deviations, and the results of T-test comparing clinical and non-clinical samples.

Variables	Clinical Sample		Non-clinical Sample, overall		Mean diff.	t (df)	*p*	Cohen’s d (95% CI)
	M	SD	M	SD				
Potentially traumatic events	5.16	3.584	3.38	2.855	-1.781	-3.300 (59.125)	0.002	-0.599 [-0.906, -0.292]
Impairment in psychosocial functioning	2.98	1.859	1.79	1.717	-1.187	-4.092 (59.972)	<.001	-0.683 [-0.996, -0.369]
Posttraumatic stress symptoms	27.10 ^1^	15.236	18.13	14.300	-8.977	-3.839 (63.680)	<.001	-0.622 [-0.928, -0.314]
Dissociation symptoms	4.12	2.331	2.69	2.023	-1.432	-3.421 (78.981)	0.001	-0.670 [-1.047, -0.290]
Moods and feelings, overall	14.50	7.186	8.64	7.109	-5.862	-5.324 (67.130)	<.001	-0.823 [-1.130, -0.515]
PTSD Checklist, overall	36.98 ^2^	20.635	19.96	19.623	-17.015	-4.503 (87.536)	<.001	-0.851 [-1.230, -0.469]
Intrusion symptoms	8.91	6.116	4.74	5.317	-4.174	-3.835 (81.197)	<.001	-0.743 [-1.119, -0.365]
Avoidance	3.84	2.820	2.28	2.590	-1.569	-3.075 (85.002)	.003	-0.587 [-0.958, -0.213]
Negative alterations in cognition and mood	14.31	8.118	7.63	7.520	-6.678	-4.523 (85.939)	<.001	-0.863 [-1.243, -0.479]
Alterations in arousal and reactivity	9.91	6.097	5.48	5.503	-4.430	-4.028 (84.089)	<.001	-0.774 [-1.151, -0.394]
Post-Traumatic Cognitions, overall	61.91 ^3^	19.789	46.26	17.057	-15.653	-5.064 (56.563)	<.001	-0.896 [-1.215, -0.576]
Permanent and disturbing change	30.48	10.989	21.40	9.406	-9.079	-5.293 (56.394)	<.001	-0.942 [-1.261, -0.621]
Fragile person in a scary world	31.43	9.446	24.86	8.480	-6.575	-4.435 (57.589)	<.001	-0.763 [-1.079, -0.445]
Social support, overall	146.67^4^	22.719	173.73	39.251	27.065	6.045 (67.022)	<.001	0.718 [0.365, 1.069]
Parental support	39.42	12.896	45.83	13.163	6.405	3.082 (59.823)	0.003	0.488 [0.170, 0.805]
Teacher support	32.78	12.279	38.95	13.016	6.175	3.103 (61.140)	0.003	0.478 [0.161, 0.795]
Classmates support	28.33	12.204	40.03	13.295	11.706	5.838 (60.343)	<.001	0.890 [0.564, 1.215]
Close Friend support	45.57	11.563	48.88	11.911	3.310	1.608 (45.486)	0.115	0.279 [-0.070, 0.627]

**^1^**Post-traumatic stress symptoms score of 21 or higher indicate screening-level probable PTSD; **^2^**On a PTSD checklist, the overall cut-off score for identifying probable PTSD is a score of 33 or higher (CATS = primary PTSD outcome; PTSD checklist = convergent validation only.); **^3^**Post-traumatic cognitions overall scores in the range of 46 to 48 (or greater) are “clinically significant” and typical of children and adolescents with PTSD; M, Mean; SD, standard deviation.

Sample comparisons showed significant differences. The clinical sample had higher scores on: exposure to PTEs, impairment in psychosocial functioning, CATS PTSD, dissociation, moods and feelings, PTSD Checklist (total), intrusion, avoidance, negative alterations in cognition and mood, arousal/reactivity, and post−traumatic cognitions (both subscales: “permanent and disturbing change”, “fragile person in a scary world”). Perceived social support was lower in the clinical group for the total score and for parent, teacher, and classmate support. Robustness check: Mann–Whitney U tests replicated all t−test differences and identified one additional significant difference—close−friend support was higher in the non−clinical sample, whereas the corresponding Welch t−test was non−significant. Screening−level criteria for probable PTSD were met by children in the clinical sample; PTE exposure had not been identified prior to hospitalization.

Furthermore, it was assumed (H2) that scores of post−traumatic stress, moods and feelings, dissociation, perceived social support, and post−traumatic cognitions in the non−clinical sample of children who had experienced more than one PTE (n = 243) would still differ significantly from the scores of children in the clinical sample (n = 50). The independent samples t−test results for the clinical and non−clinical groups (children who experienced more than one PTE) are presented in **Table 8**.

**Table 8:** Means, standard deviations, and the results of T-test comparing clinical sample and non-clinical sample of children who experienced more than one traumatic event.

Variables	Clinical Sample		Non-clinical Sample, >1 traumatic event		Mean diff.	t (df)	*p*	Cohen’s d (95% CI)
	M	SD	M	SD				
Potentially traumatic events	5.16	3.584	4.65	2.560	-0.516	-0.947(60.979)	0.347	-0.184 [-0.498, 0.130]
Impairment in psychosocial functioning	2.98	1.859	2.22	1.684	-0.755	-2.540 (65.686)	0.013	-0.439 [-0.760, -0.117]
Posttraumatic stress symptoms	27.10^1^	15.236	23.00 ^1^	13.854	-4.102	-1.713(69.502)	0.091	-0.290 [-0.604, 0.025]
Dissociation symptoms, overall	4.12	2.331	3.56	2.071	-0.564	-1.206(85.325)	0.231	-0.256 [-0.672, 0.162]
Moods and feelings, overall	14.50	7.486	10.79	7.089	-3.707	-3.260(75.597)	0.002	-0.521 [-0.835, -0.206]
PTSD Checklist, overall	36.98^2^	20.635	28.07 ^2^	20.296	-8.913	-2.077(88.866)	0.041	-0.436 [-0.850, -0.018]
Intrusion symptoms	8.91	6.116	6.67	5.812	-2.237	-1.788(88.527)	0.077	-0.375 [-0.789, 0.041]
Avoidance	3.84	2.820	3.17	2.711	-0.671	-1.156(88.661)	0.251	-0.242 [-0.654, 0.171]
Negative alterations in cognition and mood	14.31	8.118	11.07	7.353	-3.244	-1.987(87.151)	0.050	-0.419 [-0.836, 0.000]
Alterations in arousal and reactivity	9.91	6.097	7.56	5.907	-2.356	-1.861(87.912)	0.066	-0.392 [-0.809, 0.026]
Post-Traumatic Cognitions, overall	61.83	19.811	51.48 ^3^	17.207	-10.432	-3.290(62.297)	0.002	-0.588 [-0.914, -0.262]
Permanent and disturbing change	30.48	11.989	24.00	9.926	-6.476	-3.656(63.741)	0.001	-0.639 [-0.965, -0.311]
Fragile person in a scary world	31.43	9.446	27.48	8.199	-3.956	-2.614(62.234)	0.011	-0.468 [-0.792, -0.143]
Social support, overall	146.67	22.719	166.66	38.921	19.996	4.225(80.630)	<.001	0.543 [0.182, 0.902]
Parent support	39.42	12.896	43.96	13.550	4.541	2.104(68.789)	0.039	0.338 [0.012, 0.664]
Teacher support	32.78	12.279	37.07	12.618	4.290	2.097(67.650)	0.040	0.342 [0.015, 0.668]
Classmates support	28.33	12.204	37.66	13.614	9.335	4.473(69.888)	<.001	0.698 [0.364, 1.032]
Close Friend support	45.57	11.563	47.82	12.381	2.253	1.059(51.590)	0.295	0.184 [-0.173, 0.541]

**^1^**Post-traumatic stress symptoms score of 21 or higher indicates screening-level probable PTSD; **^2^**On a PTSD checklist, the overall cut-off score for identifying probable PTSD is a score of 33 or higher (CATS = primary PTSD outcome; PTSD checklist= convergent validation only); **^3^**Post-traumatic cognitions overall scores in the range of 46 to 48 (or greater) are “clinically significant” and typical of children and adolescents with PTSD; M = Mean; SD = standard deviation. Mann–Whitney U tests confirmed all Welch t−test results for the ≥1 PTE comparison; no additional significant differences emerged.

The independent‐samples t−test revealed significant differences between the clinical sample and the non−clinical sample exposed to >1 PTE. As pre−specified, CATS PTSD symptoms served as the primary PTSD outcome; PTSD Checklist (PLC-5) results are presented only as convergent validation. The clinical sample showed higher PTSD Checklist total scores; however, in the non−clinical subsample, a CATS PTSD symptoms score ≥ 21 indicates screening−level probable PTSD (not a clinical diagnosis). The non−clinical sample also showed lower impairment in psychosocial functioning, negative moods and feelings, overall post−traumatic cognitions, and both posttraumatic cognitions factors (“permanent and disturbing change”, “fragile person in a scary world”). Nevertheless, mean CPTCI scores in the non−clinical >1 PTE sample exceeded commonly used clinical ranges (total > 48), indicating clinically meaningful cognitions despite lower overall symptom burden. No significant differences emerged for the number of PTEs, CATS PTSD symptoms total, dissociation, PTSD Checklist (intrusion, avoidance, negative cognitions/mood, arousal), nor for close−friend support. Together, these findings suggest high screening−level PTSD risk in the non−clinical >1 PTE sample and more severe cognitive burden in the clinical sample.

In terms of perceived social support, samples differed. Children in the non−clinical sample reported higher overall perceived support than children in the clinical sample. This pattern also held for teacher support and classmate support, indicating stronger school−based relationships in the non−clinical ample. Parental support was likewise higher in the non−clinical group. Together, these between−sample differences indicate that children in community settings perceive more support from parents, teachers, and classmates. We interpret these findings as associations rather than effects.

### 3.3 Correlation analysis in clinical and non-clinical samples

To test H3, we examined associations between exposure to PTEs and psychological outcomes (PTSD symptoms, dissociation, mood, post−traumatic cognitions) and perceived social support, using Pearson correlations in each sample. Hypothesis H4 focused specifically on social support. To test H4, we examined how different forms of perceived social support (parents, teachers, classmates, close friends) relate to psychological outcomes (PTSD symptoms, dissociation, mood, post−traumatic cognitions) within each sample, using Pearson correlations.

The results of the correlation analysis in the clinical sample are presented in **Table 9**.

**Table 9:** Correlations between the study variables in the clinical sample.

	1	2	3	4	5	6	7	8	9	10	11	12	13	14	15	16	17
1. Potentially traumatic events	—																
2. Impairment in psychosocial functioning	.510***	—															
3.Posttraumatic stress symptoms	.631***	.430**	—														
4. Dissociation symptoms	.604***	.166	.468**	—													
5. Moods and feelings, overall	.461**	.434**	.755***	.417**	—												
6. PTSD Checklist, overall	.591***	.536***	.842***	.398**	.681***	—											
7. Intrusion symptoms	.577***	.494**	.775***	.305*	.597***	.877***	—										
8. Avoidance	.545***	.376*	.716***	.438**	.614***	.808***	.725***	—									
9. Negative alterations in cognition and mood	.522***	.556***	.767***	.360*	.667***	.936***	.713***	.722***	—								
10. Alterations in arousal and reactivity	.477**	.403**	.721***	.348*	.533***	.886***	.678***	.583***	.786***	—							
11. Post-Traumatic Cognitions	.556***	.397**	.819***	.406**	.755***	.878***	.766***	.663***	.837***	.781***	—						
12. Permanent and disturbing change	.524***	.422**	.799***	.394**	.745***	.860***	.753***	.675***	.839***	.727***	.973***	—					
13. Fragile person in a scary world	.555***	.340*	.786***	.392**	.716***	.839***	.730***	.605***	.778***	.792***	.963***	.875***	—				
14. Social support, overall	-.178	-.026	.034	-.035	-.141	.063	-.015	-.032	.018	.227	-.020	-.054	.023	—			
15. Parent support	.051	-.109	.128	.131	-.067	.082	.066	.101	.018	.142	.057	.018	.099	.274	—		
16. Teacher support	.059	-.080	.137	.118	-.014	.210	.183	.228	.150	.223	.290	.256	.310*	.534**	.264	—	
17. Classmates support	.183	.113	.135	.196	.185	.167	.181	.088	.136	.158	.123	.121	.116	.503**	.065	.302*	—
18. Close Friend support	-.003	.171	.074	-.009	-.110	.212	.014	-.066	.254	.396*	-.005	-.018	.012	.727***	-.114	.164	.422*

*p <.05, **p <.01, ***p <.001.

In the clinical sample, exposure to PTEs were strongly associated with impairment in psychosocial functioning (r = .510, p <.001), post−traumatic stress symptoms (r = .631, p <.001), dissociation symptoms (r = .604, p <.001), and the overall PTSD Checklist score (r = .591, p <.001). Exposure to PTEs were also moderately associated with moods and feelings (r = .461, p <.01). In addition, exposure to PTEs showed strong associations with post−traumatic cognitions (r = .556, p <.001), including viewing oneself as a fragile person in a dangerous world (r = .555, p <.001) and beliefs about permanent and disturbing change (r = .524, p <.001). A small but statistically significant association was observed between teacher support and the perception of oneself as a fragile person in a dangerous world (r = .310, p <.05). Close−friend support was also significantly associated with alterations in arousal and reactivity (r = .396, p <.05). No other significant associations with perceived social support were found in the clinical sample.

The results of the correlation analysis in the overall non-clinical sample are presented in **Table 10**.

**Table 10:** Correlations between the study variables in the non-clinical sample.

	1	2	3	4	5	6	7	8	9	10	11	12	13	14	15	16	17
1. Potentially traumatic events	—																
2. Impairment in psychosocial functioning	.435***	—															
3.Posttraumatic stress symptoms	.615***	.627***	—														
4. Dissociation symptoms	.639***	.447***	.839***	—													
5. Moods and feelings, overall	.486***	.538***	.788***	.643***	—												
6. PTSD Checklist, overall	.666***	.562***	.899***	.791***	.720***	—											
7. Intrusion symptoms	.608***	.468***	.840***	.699***	.609***	.927***	—										
8. Avoidance	.579***	.514***	.774***	.661***	.647***	.873***	.807***	—									
9. Negative alterations in cognition and mood	.669***	.536***	.884***	.804***	.721***	.961***	.834***	.787***	—								
10. Alterations in arousal and reactivity	.658***	.577***	.822***	.736***	.710***	.946***	.824***	.783***	.884***	—							
11. Post-Traumatic Cognitions	.564***	.619***	.833***	.808***	.812***	.903***	.794***	.784***	.911***	.845***	—						
12. Permanent and disturbing change	.536***	.571***	.812***	.773***	.781***	.902***	.797***	.759***	.899***	.865***	.958***	—					
13. Fragile person in a scary world	.524***	.611***	.776***	.765***	.767***	.814***	.713***	.733***	.834***	.741***	.949***	.819***	—				
14. Social support, overall	-.372***	-.363***	-.391***	-.272*	-.406***	-.290*	-.177	-.185	-.350**	-.296*	-.389***	-.385***	-.355***	—			
15. Parent support	-.347***	-.388***	-.376***	-.453***	-.428***	-.510***	-.375**	-.397***	-.545***	-.524***	-.421***	-.411***	-.391***	.759***	—		
16. Teacher support	-.268***	-.307***	-.238***	-.246*	-.277***	-.273*	-.168	-.131	-.326**	-.305**	-.202**	-.213***	-.169**	.732***	.407***	—	
17. Classmates support	-.281***	-.261***	-.344***	-.237*	-.330***	-.187	-.100	-.148	-.260*	-.148	-.367***	-.336***	-.365***	.806***	.466***	.441***	—
18. Close Friend support	-.228***	-.163**	-.235***	.021	-.205**	.061	.093	.105	.018	.052	-.211***	-.226***	-.174**	.757***	.441**	.363***	.546***

*p <.05, **p <.01, ***p <.0.

In the non−clinical sample, exposure to PTEs were strongly associated with post−traumatic stress symptoms (r = .615, p <.001), dissociation symptoms (r = .639, p <.001), the PTSD Checklist total score (r = .666, p <.001), and post−traumatic cognitions (r = .564, p <.001). Exposure to PTEs were also moderately associated with moods and feelings (r = .486, p <.001). Post−traumatic cognitions showed strong associations with post−traumatic stress symptoms (r = .833, p <.001), dissociation symptoms (r = .808, p <.001), moods and feelings (r = .812, p <.001), and the PTSD Checklist total score (r = .903, p <.001).

Perceived social support showed consistent associations with study variables. Overall social support was moderately and negatively associated with moods and feelings (r = –.406, p <.001). Parental support showed moderate negative associations with dissociation symptoms (r = –.453, p <.001), moods and feelings (r = –.428, p <.001), post−traumatic cognitions (r = –.421, p <.001), and the PTSD Checklist total score (r = –.510, p <.001). Parental support was also moderately and negatively associated with negative alterations in cognition and mood (r = –.545, p <.001) and with alterations in arousal and reactivity (r = –.524, p <.001).

## 4 Discussion

This study compared trauma exposure and trauma−related psychological outcomes between clinical and non−clinical Lithuanian children. The findings showed that hospitalized children reported higher exposure to potentially traumatic events and more severe symptoms across PTSD, dissociation, mood, and post−traumatic cognitions, which is consistent with earlier work showing elevated trauma burden among clinically referred youth (4, 6, 34, 61),. Children in the non−clinical sample perceived more social support from parents, teachers, and classmates, in line with evidence that supportive environments relate to better psychological adjustment in trauma−exposed children (62, 63). The high trauma−exposure rate observed in the non−clinical sample (82.9%) should be interpreted with caution, as broad PTE checklists, anonymous self−report formats, and cultural reporting norms in Lithuania may contribute to elevated prevalence estimates.

When the comparison was restricted to children who had been exposed to more than one PTE, several differences between the samples became smaller. PTSD symptoms, dissociation, and several PTSD Checklist clusters did not differ significantly, echoing studies showing that repeated or cumulative trauma in community settings can be associated with symptom levels similar to those seen in clinical samples (1, 93). However, hospitalized children continued to show higher levels of negative mood and more intense post−traumatic cognitions, including beliefs about permanent change and fragility in a dangerous world, which aligns with research on the role of trauma−related beliefs in symptom maintenance (4, 41). Children in the non−clinical multiple−PTE group still reached clinically meaningful ranges on post−traumatic cognitions, consistent with findings that maladaptive trauma−related beliefs can emerge even outside clinical environments (87, 94). These children perceived higher levels of support from teachers, classmates, and parents, reflecting the known role of school and family relationships as important protective resources (95, 96).

Importantly, most group differences were not only statistically significant but also showed medium-to-large effect sizes (Cohen’s d), indicating that the magnitude of these differences is meaningful at the practical and clinical levels. As this study is cross−sectional, all associations should be interpreted as non−causal, and the temporal ordering between trauma exposure, symptoms, and social support cannot be determined. Across both samples, exposure to PTEs was associated with PTSD symptoms, dissociation, negative mood, and post−traumatic cognitions. These associations follow previous evidence indicating strong links between trauma exposure and psychological difficulties in both clinical and community children samples (97–99). Post−traumatic cognitions were strongly associated with PTSD symptoms, dissociation, and negative mood, consistent with cognitive frameworks emphasizing the role of trauma−related beliefs in shaping children’s emotional and behavioral responses (100, 101). These findings highlight the need for future studies to examine post−traumatic cognitions in greater depth, as cognitive appraisals appear to play a central role across both clinical and community samples.

The patterns of social support differed across the groups. In the clinical sample, support showed weak and sometimes unexpected associations. Teacher support had a small positive link with vulnerability−related cognitions, and close friend support correlated with arousal symptoms. These findings may reflect heightened emotional and physiological reactivity among trauma−exposed hospitalized children, which is consistent with recent evidence showing increased stress reactivity during interpersonal interactions in youth with PTSD (102). In addition, recent meta−analytic evidence indicates that trauma−focused treatments for children and adolescents can improve aspects of social and interpersonal functioning, although effects are generally small and heterogeneous (103). In contrast, social support in the non−clinical sample demonstrated clearer protective associations. Higher parental support was linked with fewer dissociative symptoms, lower negative mood, less severe PTSD symptoms, and more adaptive cognitions, aligning with findings that parental support acts as an important regulatory and buffering resource (65, 104). Teachers appeared to have a meaningful role, particularly for children in community settings. Conceptualizing teachers as quasi−attachment figures has been supported in recent work suggesting that teacher–student relationships may provide stability, predictability, and emotional regulation cues in everyday school environments (66, 105). This perspective helps explain why teacher support was consistently higher in non−clinical children and may offer a valuable direction for strengthening trauma−sensitive school practices. Consistent with this, a recent meta−analysis shows that group−based PTSD interventions for youth—often delivered in school or community settings—produce medium pooled effects on symptom reduction (106).

This study contributes several new insights. It provides the first systematic comparison of psychiatric inpatient and general−population children in Lithuania with regard to trauma exposure and trauma−related outcomes. The results revealed a high amount of previously unrecognized trauma in the clinical sample, supporting concerns about under−identification of trauma in psychiatric services (19). The finding that children in the non−clinical multiple−PTE group also reached clinically significant thresholds on several measures aligns with cross−cultural research indicating substantial trauma exposure outside clinical settings (23, 25). This study also extends previous work by examining distinct sources of social support—parents, teachers, classmates, and close friends—using a multidimensional measure with strong psychometric grounding (89). The results further highlight the importance of trauma−related cognitions in both samples, consistent with research on cognitive models of trauma (101, 107).

Because these findings reflect the specific cultural and institutional context of Lithuania, including local reporting norms, family patterns, and school−based support systems, their generalizability to other countries should be considered with caution.

### 4.1 Implications and future directions

This study shows that many hospitalized children displayed screening−level PTSD, although none had been previously identified as having trauma−related difficulties. This highlights a gap in trauma recognition in Lithuanian child psychiatric inpatient care and the need for routine trauma−informed assessment. Children in the community who experienced multiple traumatic events also showed elevated risk, including clinically meaningful post−traumatic cognitions. Although their overall symptoms were lower than in the clinical group, they still require early screening and support.

Social support patterns further emphasize the importance of family− and school−based interventions. In the clinical sample, associations with support were weak. In the community sample, higher parental and teacher support related to fewer trauma−related difficulties. These findings suggest that strengthening supportive relationships in families and schools could be relevant for reducing vulnerability and supporting children’s well−being. Overall, the results support several priorities:

1. Implementing systematic trauma screening in child psychiatric services,.
2. Integrating trauma−informed assessment protocols,.
3. And reinforcing school−based and family−centered support systems to ensure early recognition and timely intervention.

### 4.2 Limitations of the study

This study has several limitations that should be considered when interpreting the findings. The clinical sample was relatively small (n = 50) and diagnostically heterogeneous, reflecting the natural patient population of the Child Psychiatry Department at Vilnius University Hospital Santaros Klinikos. Although this approach allowed us to capture real−world variability, diagnostic heterogeneity may have affected the patterns of associations. In the non−clinical sample, only about 19% of invited participants agreed to take part, raising concerns about self−selection and limited representativeness. The unequal group sizes between clinical and community samples may also have influenced group comparisons.

The study relied on a cross−sectional design, which restricts conclusions to non−causal associations and prevents determining the temporal order between PTEs and psychological outcomes. All measures were based on self−report, which may introduce response bias. No diagnostic interviews were conducted; therefore, PTSD results represent screening−level indicators rather than clinical diagnoses. The PCL−5, originally designed for adults, was used only for convergent validation and to supplement child−appropriate measures; this application has inherent limitations when used with adolescents.

The CASSS Level 1 was administered across the full 10–17 age range to maintain a single instrument during mixed−age administration. Although CASSS Level 1 has strong psychometric support and substantial item overlap with Level 2, it was originally intended for younger grades, and this broader use requires cautious interpretation. Additionally, key demographic factors such as age and gender were recorded but not examined as potential moderators. These variables may influence trauma exposure and psychological responses.

Several constructs examined in this study—PTSD symptoms, dissociation, mood, and social support—are interrelated. Our analyses focused on group comparisons and bivariate correlations, without applying multivariable models to account for overlapping variance. Future research should employ larger and more balanced samples, integrate structured diagnostic interviews, and apply multivariable or logistic regression approaches to identify unique predictors of trauma−related outcomes. Finally, because the study was conducted in Lithuania, cultural factors—including reporting norms, family dynamics, and school environments—may have influenced the results, and generalization to other contexts should be made cautiously.

## 5 Conclusions

This study showed clear differences between clinical and non−clinical children. Hospitalized children had higher trauma exposure and more severe trauma−related symptoms, including dissociation, negative mood, and post−traumatic cognitions. Many displayed screening−level PTSD that had not been identified before hospitalization. Children in the community who had experienced more than one potentially traumatic event also demonstrated a heightened risk—particularly in trauma−related thinking patterns—highlighting the importance of early screening even outside clinical environments. Social support patterns differed between groups. Support was weaker in the clinical sample, while higher parental and teacher support in the non−clinical group was associated with fewer trauma−related difficulties.

Overall, the findings point to the importance of systematic trauma identification, trauma−informed assessment in psychiatric services, and stronger school− and family−based support systems to improve recognition and response to children’s trauma−related needs.

## Data Availability

The raw data supporting the conclusions of this article will be made available by the authors, without undue reservation.
